# Combined Methylation and Transcriptome Analysis of Liver Injury of Nonalcoholic Fatty Liver Disease Induced by High Alcohol-Producing Klebsiella pneumoniae

**DOI:** 10.1128/spectrum.05323-22

**Published:** 2023-04-06

**Authors:** Rui Zhang, Ziying Xu, Guanhua Xue, Junxia Feng, Bing Du, Lin Gan, Zheng Fan, Tongtong Fu, Yanling Feng, Hanqing Zhao, Jinghua Cui, Chao Yan, Xiaohu Cui, Ziyan Tian, Jinfeng Chen, Zihui Yu, Jing Yuan

**Affiliations:** a Capital Institute of Pediatrics, Beijing, China; b Children's Hospital Capital Institute of Pediatrics, Chinese Academy of Medical Sciences & Peking Union Medical College, Beijing, China; Shenzhen Bay Laboratory

**Keywords:** HiAlc *Kpn*, endogenous ethanol, DNA methylation, liver injury

## Abstract

It has been known that high alcohol-producing Klebsiella pneumoniae (HiAlc *Kpn*) is one of causative agents of nonalcoholic fatty liver disease (NAFLD). However, how HiAlc *Kpn* promotes liver injury remains unclear. Recent findings suggest that DNA methylation might associate with the pathogenesis of NAFLD. Herein, the role of DNA methylation in HiAlc *Kpn*-induced liver injury was investigated. Murine models of NAFLD were established in C57BL/6N wild-type mice by gavaging HiAlc *Kpn* for 8 weeks. The liver injury was assessed based on the liver histopathology and biochemical indicators. In addition, DNA methylation in hepatic tissue was assessed by using dot bolt of 5-mC. RNA sequencing analysis and whole-genome bisulfite sequencing (WGBS) analysis were also performed. HiAlc *Kpn* significantly increased the activity of aspartate transaminase (AST), alanine transaminase (ALT), triglycerides (TGs), and glutathione (GSH), while hypomethylation was associated with liver injury in the experimental mice induced by HiAlc *Kpn*. The GO and KEGG pathway enrichment analysis of the transcriptome revealed that HiAlc *Kpn* induced fat metabolic disorders and DNA damage. The conjoint analysis of methylome and transcriptome showed that hypomethylation regulated related gene expression in signal pathways of lipid formation and circadian rhythm, including *Rorα* and *Arntl1*genes, which may be the dominant cause of NAFLD induced by HiAlc *Kpn*. Data suggest that DNA hypomethylation might play an important role in liver injury of NAFLD induced by HiAlc *Kpn*. Which possibly provides a new sight for understanding the mechanisms of NAFLD and selecting the potential therapeutic targets.

**IMPORTANCE** High alcohol-producing Klebsiella pneumoniae (HiAlc *Kpn*) is one of causative agents of nonalcoholic fatty liver disease (NAFLD) and could induce liver damage. DNA methylation, as a common epigenetic form following contact with an etiologic agent and pathogenesis, can affect chromosome stability and transcription. We conjointly analyzed DNA methylation and transcriptome levels in the established murine models to explore the potential mechanisms for further understanding the role of DNA methylation in the liver damage of HiAlc *Kpn*-induced NAFLD. The analysis of the DNA methylation landscape contributes to our understanding of the entire disease process, which might be crucial in developing treatment strategies.

## INTRODUCTION

Nonalcoholic fatty liver disease (NAFLD) is a common chronic liver disease, with a prevalence of 15 to 30% globally (1, 2). In the past decade, there has been a tremendous increase in NAFLD incidence in Europe, China, India, the United States, and other regions (3), making NAFLD a global health problem. NAFLD patients are characterized by hepatocellular ballooning and may progress to more serious status such as nonalcoholic steatohepatitis (NASH), cirrhosis, or hepatocellular carcinoma (4). The pathogenesis of NAFLD is multifactorial, mainly mediated by heritable and environmental risk factors such as alcohol (1). In addition, our previous study has shown that HiAlc *Kpns*, strains of high alcohol-producing Klebsiella pneumoniae, isolated from clinical fecal samples from a patient with an auto-brewery syndrome (ABS)/nonalcoholic steatohepatitis (NASH) produced alcohol by fermentation in the intestinal tract and induced NAFLD in mice (5). However, the mechanism of liver damage in NAFLD mice induced by HiAlc *Kpn* remains unclear.

DNA methylation, as a common epigenetic form following contact with an etiologic agent and pathogenesis, can affect embryonic development, chromatin structure, X-chromosome inactivation, genomic imprinting, chromosome stability, and transcription (6). It has been well known that transcription will be repressed when the promoter regions of genes present hypermethylation, while transcription will be activated when gene body regions present hypermethylation (7–9). It has been also shown that aberrant DNA methylation is associated with various cancers, including hepatocellular carcinoma (6, 10), while hypomethylation leading to genomic instability in tumor cells is a hallmark of tumor cells (11). In addition, DNA methylation in liver, lung, and brain tissues is not constant but varies with biological rhythms (12–14). It has been reported that methyl-depleted diets may enhance steatohepatitis, cirrhosis, and hepatocellular carcinoma in murine models (15). Although DNA methylation might be a potent mechanism for regulating gene expression and maintaining genome stability in many diseases, its potential role in the development and progression of NAFLD induced by HiAlc *Kpn* in mice is still unclear.

Therefore, the present study aimed to elucidate the epigenetic landscape of liver damage and find functionally relevant methylation differences. For these purposes, we analyzed DNA methylation and RNA sequencing of liver tissues of the experimental mice compared with controls. Our data indicated that DNA methylation could regulate biological rhythms and the disease process of NAFLD mice induced by HiAlc *Kpn*.

## RESULTS

### HiAlc Klebsiella induced liver injury and DNA hypomethylation in NAFLD mice model.

**FIG 1:**
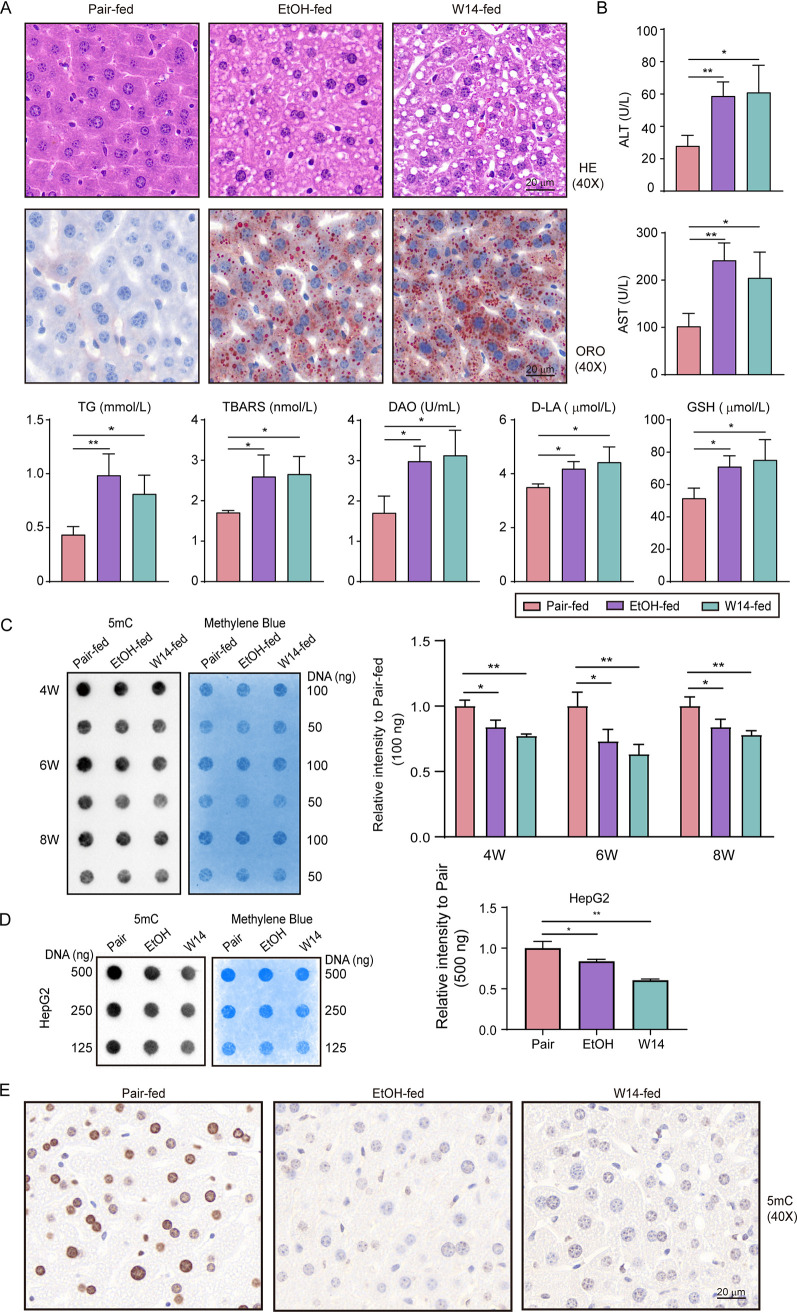
HiAlc*Kpn* (W14) induced liver injury and DNA hypomethylation in NAFLD mice. (A) Liver histology for the assessment of hepatic steatosis induced by HiAlc *Kpn* W14 for 8 weeks visualized by H&E (40×) and Oil Red O staining (40×). (B) Biochemical indicators (ALT, AST, TG, TBARS, and GSH) of the liver injury induced by HiAlc *Kpn* W14 in mice for 8 weeks. (C) The 5-mC-specific dot blot. gDNA was isolated from liver tissues, and 100 ng and 50 ng of gDNA were loaded per dot. Dot intensity analysis by the ImageJ is shown on the right. (D) The 5-mC-specific dot blot. The liver cells (HepG2) were treated by EtOH or the culture supernatant of HiAlc *Kpn* for 48 h, respectively. The cells were collected and gDNA was extracted for the dot blot experiment. A total of 500 ng (top), 250 ng (middle), and 125 ng (bottom) of gDNA were loaded per dot. Dot intensity analysis by the ImageJ is shown on the right. (E) Immunohistochemical analysis of 5-mC levels in sections of the liver tissues of mice for 8 weeks feeding. All experiments were performed in triplicates, and data represent mean ± SD. *n* = 6, *, *P* < 0.05; **, *P* < 0.01.

To verify whether the liver injury of NAFLD induced by HiAlc *Kpn* is associated with DNA methylation level, pathological indicators, biochemical indicators, immunohistochemical, and dot blot analysis of liver tissue were measured in the experimental mice. The results showed that there were severe pathological changes and fat accumulation in the livers of W14-fed mice after gavage for 8 weeks, which were similar as the EtOH-fed group, but not in the pair-fed mice (Fig. 1A). Both W14-fed and EtOH-fed mice had significantly increased levels of AST and ALT in the serum, and TG, TBARS (Thiobarbituric acid reactive substance), and GSH in the liver (Fig. 1B), suggesting that there were pathophysiological changes in their livers. Simultaneously, the 5mC levels in W14-fed and EtOH-fed mice were significantly decreased from week 4 to 8 (Fig. 1C). Thus, mice gavaged with W14 for 8 weeks were used for the present study. In addition, the DNA hypomethylation of liver cells (HepG2) treated by EtOH and culture supernatant of HiAlc *Kpn* was determined by dot blot (Fig. 1D). The immunohistochemistry (IHC) images were also consistent with the dot blot of the 5mC expression levels in the liver induced by W14 for 8 weeks (Fig. 1E).

### Transcriptome analysis of liver tissue of mice.

**FIG 2:**
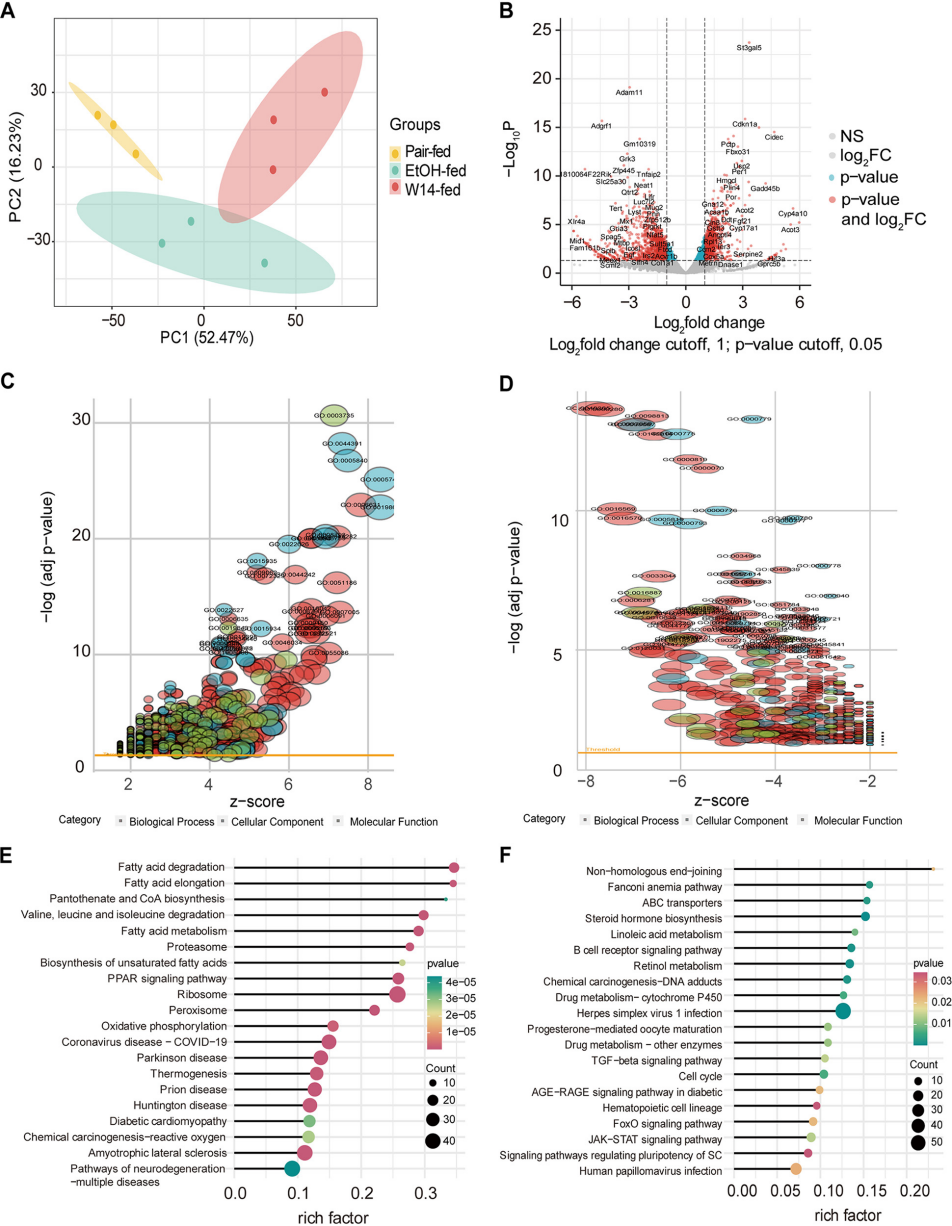
Transcriptome analysis of liver tissues of EtOH-fed mice. (A) Correlation analysis of differentially expressed genes (DEGs) of liver tissues from pair-, EtOH-, and W14-fed mice. (B) Volcano plot of DEGs analysis between EtOH- and pair-fed mice. Gene Ontology (GO) enrichment analysis of upregulated (C) and downregulated (D) DEGs in the liver tissues between EtOH- and pair-fed mice. Kyoto Encyclopedia of Genes and Genome (KEGG) pathway enrichment analysis of upregulated (E) and downregulated DEGs (F) in liver tissues of EtOH- and pair-fed mice.

The principal-component analysis (PCA) showed high intragroup repeatability of liver tissue of pair-, EtOH-, and W14-fed mice gavaged for 8 weeks (Fig. 2A). Analysis of DEseq2 showed the differentially expressed genes (DEGs) among the three groups. Volcano plot of the DEGs revealed that 871 genes were upregulated, and 1,188 genes were downregulated in EtOH-fed mice compared with pair-fed mice (Fig. 2B). Analysis of GO and KEGG pathways revealed that upregulated DEGs were involved in the biosynthesis of unsaturated fatty acids and fatty acid elongation pathways, implying that there was activation of the fat deposition signaling pathway in the EtOH group (Fig. 2C and E). In addition, downregulated DEGs in the EtOH group, relative to the pair-fed group, were enriched in histone methylation, histone lysine methylation, peptidyl-lysine methylation, DNA repair, Fanconi anemia pathway, and hematopoietic cell lineage pathways, suggesting that there were epigenetics changes and EtOH induced DNA damage (Fig. 2D and F).

**FIG 3:**
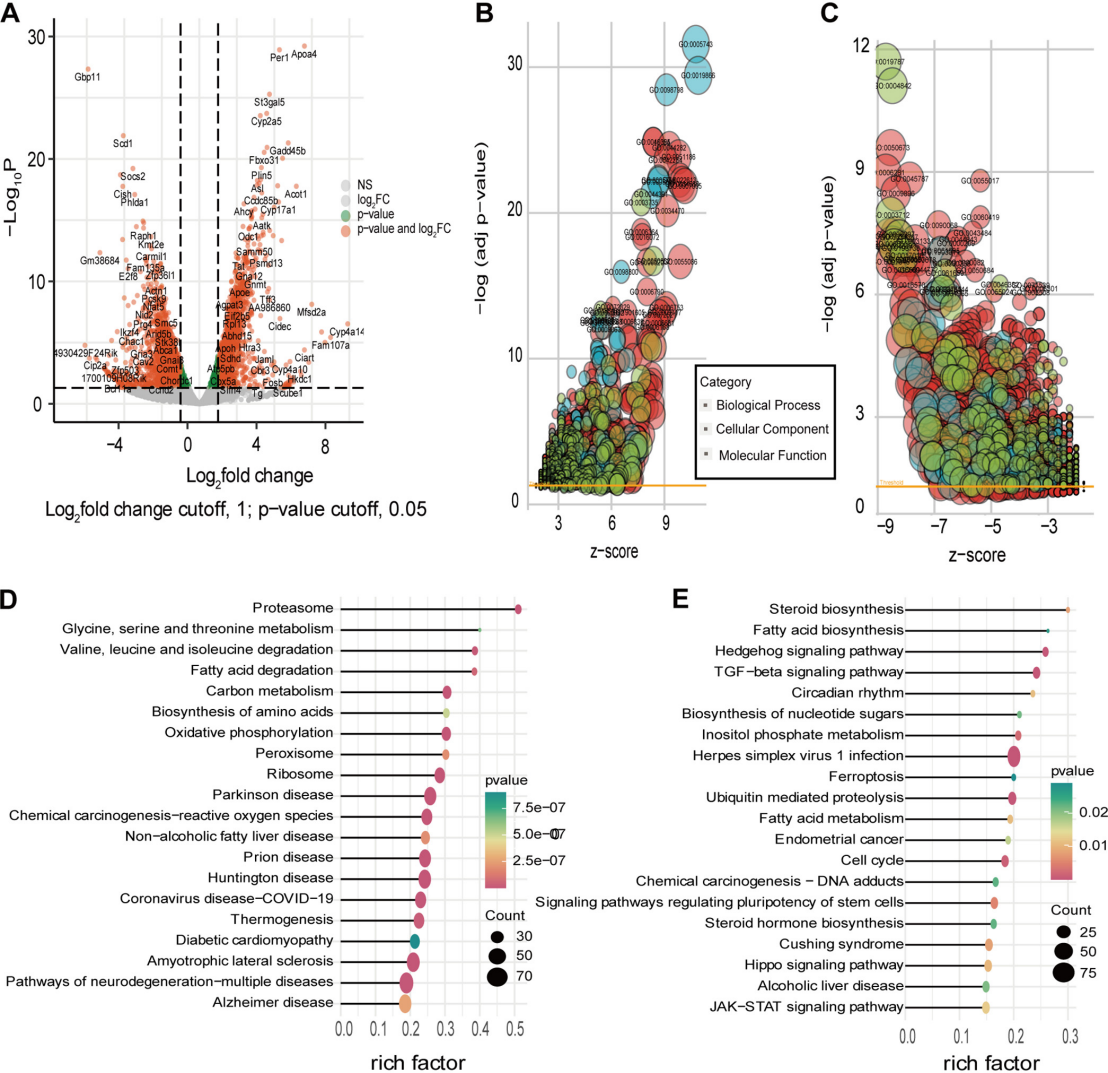
Transcriptome analysis of liver tissues of mice with W14-induced NAFLD. (A) Volcano plot of DEGs between W14- and pair-fed mice. GO enrichment analysis of upregulated (B) and downregulated (C) DEGs in liver tissues of W14- and pair-fed mice. KEGG pathway enrichment analysis of upregulated (D) and downregulated DEGs (E) in liver tissues of W14- and pair-fed mice.

Compared to the pair-fed group, 1,996 genes were upregulated, and 2,017 genes were downregulated in the W14-fed group (Fig. 3A). Based on the GO and KEGG pathways analyses, the upregulated genes were enriched in the nonalcoholic fatty liver disease, fatty acid metabolic process, and chemical carcinogenesis − reactive oxygen species (ROS) pathways, suggesting that W14 induced nonalcoholic fatty liver disease and caused metabolic disorders (Fig. 3B and D). The downregulated DEGs in the W14 group relative to the pair-fed group, were enriched in the DNA repair, postreplication repair, lipid modification, and fatty acid metabolism pathways, suggesting that W14 induced fat metabolism disorder and DNA damage (Fig. 3C and E).

### Integration of GO enrichment analysis of liver tissue in NAFLD mice induced by HiAlc *Kpn* and EtOH.

**FIG 4:**
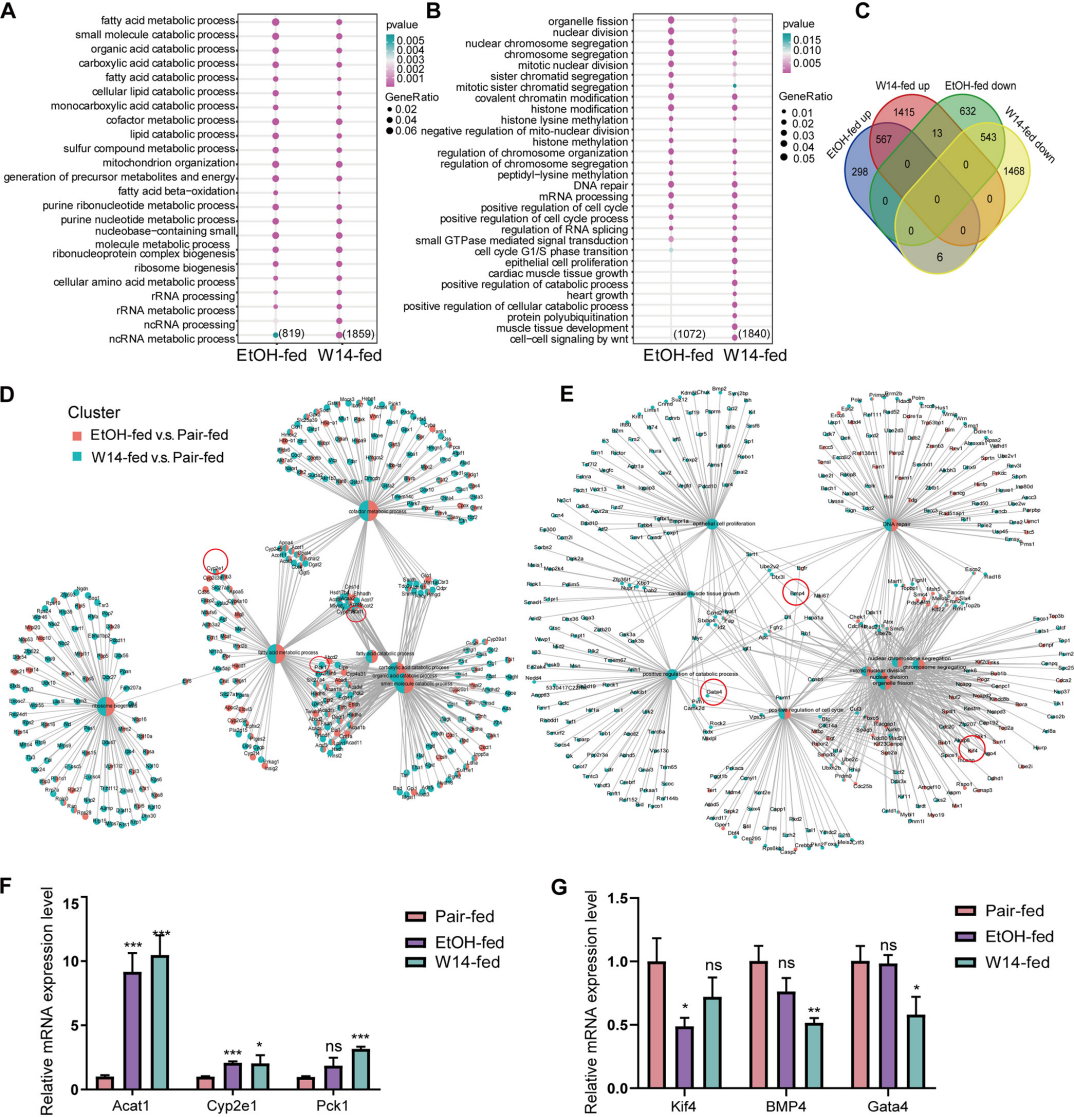
Integrative GO enrichment analysis of DEGs in liver tissues of mice with NAFLD induced by HiAlc *Kpn* and EtOH relative to the pair-fed mice. Integrative of GO enrichment analysis of upregulated (A) and downregulated (B) DEGs between W14- and EtOH-fed mice relative to the pair-fed mice. (C) Venn diagram showing the overlap of upregulated and downregulated DEGs between W14- and EtOH-fed mice compared to the pair-fed mice. List of upregulated (D) and downregulated (E) DEGs between W14- and EtOH-fed mice compared to the pair-fed mice. (F and G) RT-qPCR results showing the relative expression of upregulated (F) and downregulated (G) DEGs. These data represent the mean ± SD of three separate experiments. *n* = 3; *, *P *< 0.05; **, *P* < 0.01; ***, *P* < 0.001; ns, no statistical significance.

After analysis of the transcriptome of liver tissues of W14 or EtOH group, respectively, integration of GO enrichment analysis of DEGs in liver tissue of NAFLD mice induced by W14 and EtOH was performed. Compared to pair-fed mice, GO enrichment analysis of upregulated DEGs in the liver tissues of mice with NAFLD revealed that the fatty acid metabolic process and ribosome-related processes were enriched in the W14-fed and EtOH-fed mice, respectively (Fig. 4A). In addition, chromosome segregation, DNA repair and epigenetic modifications, including histone lysine methylation, histone methylation, and peptidyl-lysine methylation pathways, were downregulated in the W14-fed mice (Fig. 4B). A Venn diagram revealed that 567 upregulated and 543 downregulated DEGs overlapped between W14-fed and EtOH-fed mice, respectively (Fig. 4C). The DEGs of upregulated and downregulated between W14-fed and EtOH-fed mice were presented, respectively (Fig. 4D and E). The enriched pathways by the upregulated and downregulated overlapping DEGs in liver tissues between W14-fed and EtOH-fed mice are presented in Fig. S1A and S1B. To verify the transcriptome sequencing results and further analyze the changes in pathways related to fat metabolism, six representative genes were selected for real-time quantitative PCR (RT qPCR) verification. The real-time PCR further revealed that the relative expression of lipid signaling pathways or DNA damage-related genes, including *acat1*, *pck1*, and *cyp2e1* (cytochrome P450 family 2 subfamily E member 1), was elevated in the liver tissues of W14-fed mice, while the transcription factor related genes, including *kif4*, *bmp4*, and *gata4*, were decreased (Fig. 4F and G). The trend in expression of these genes was consistent with the RNA-seq results, implying that the RNA sequencing data reliably reflected the change in gene expression.

### DNA methylation changes in the liver tissues of HiAlc Klebsiella-induced NAFLD mice.

**FIG 5:**
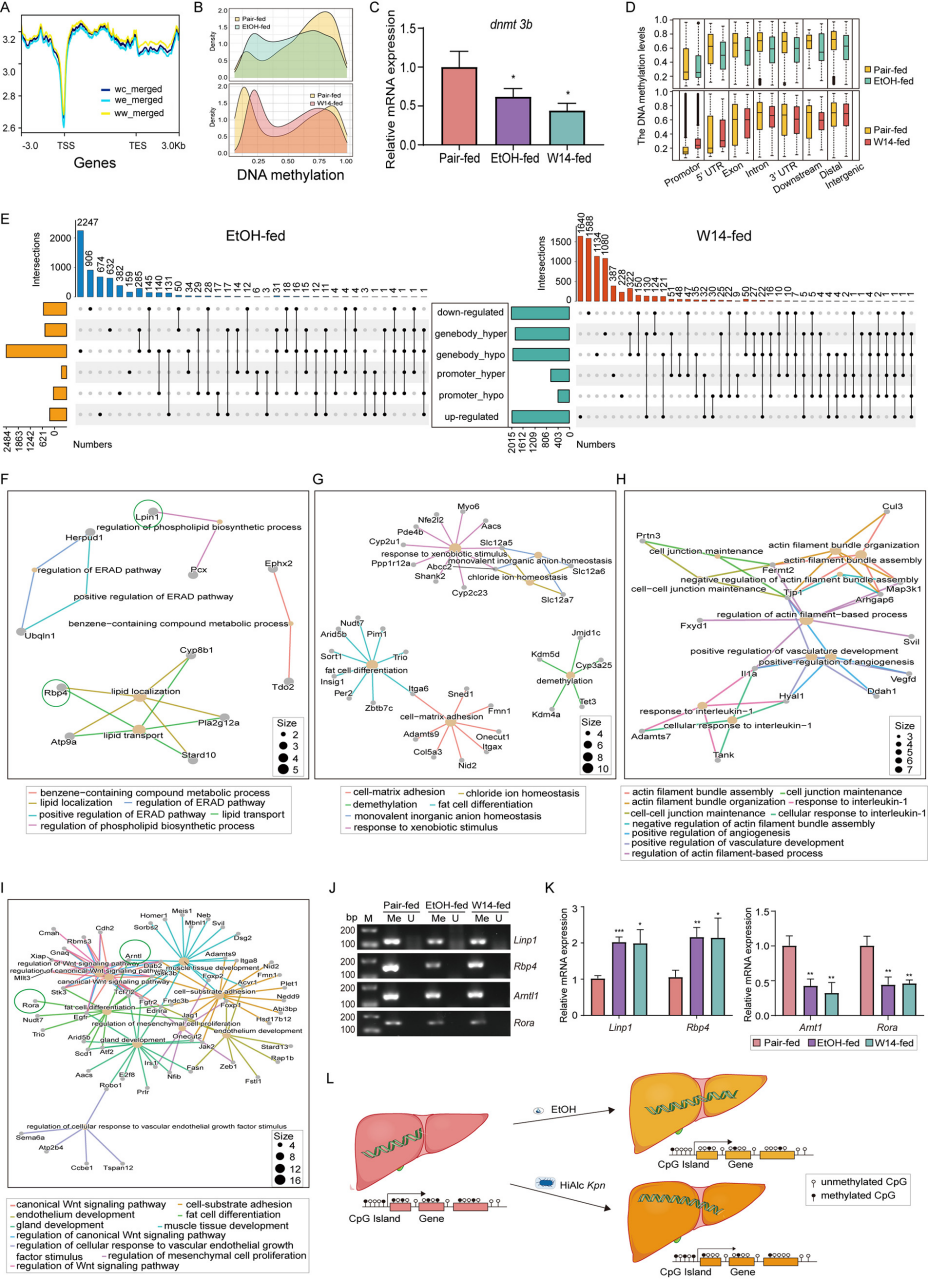
Integrative global methylation and transcriptome analyses. (A) The overall DNA methylation levels in pair-, EtOH-, and W14-fed mice were shown as wc_merged, we_merged, and ww_merged, respectively. (B) The whole DNA methylation level in EtOH- (top) and W14-fed mice (bottom), relative to the pair-fed mice. (C) The mRNA of *dnmt3b* was verified by real-time qPCR. (D) The DNA methylation level of DMRs at indicated genomic regions in EtOH- (top) and W14-fed mice (bottom), compared to the pair-fed mice. (E) The visualized upset image of DNA methylation and transcriptome data from EtOH-fed mice (left) and W14-fed mice (right), compared to the pair-fed mice. GO pathways enrichment of promoter hypomethylation associated with the significantly upregulated DEGs (F) and gene-body hypomethylation associated with downregulated DEGs (G) in EtOH-fed mice, compared to the pair-fed mice. GO pathways enrichment of promoter hypermethylation associated with the significantly downregulated DEGs (H) and gene-body, except for 5’UTR hypomethylation associated with downregulated DEGs (I) in W14-fed mice, compared to the pair-fed mice. (J) Agarose gel electrophoresis of methylation-specific PCR of *Linp1*, *Rbp4*, *Arntl1*, and *Rorα* genes (Me, Methylation primer; U, un-Methylation primer). (K) RT-qPCR results showing the relative expression of upregulated (left) and downregulated (right) genes. The data represent the mean ± SD of three separate experiments. *n* = 3; *, *P *< 0.05; **, *P* < 0.01; ***, *P* < 0.001. (L) Schematic diagram of DNA methylation in mice with NAFLD induced by W14.

To further determine whether gene expression is associated with the methylation level or the distribution of DNA methylation in NAFLD mice induced by W14, methylation profiles were performed according to gene structure. The analysis of the global levels of methylation revealed that most of the transcription start sites (TSS) and transcription end sites (TES) were normally unmethylated, and overall hypomethylated at the TSS and TES in NAFLD mice induced by W14 or EtOH, compared to the pair-fed mice (Fig. 5A). The analysis of the methylation levels of global or gene elements, including promoter, 5′UTR, exon, intron, and 3′UTR regions, revealed an overall hypomethylation in EtOH-fed mice, compared to pair-fed mice (Fig. 5B and D), nevertheless, in the W14-fed mice, most regions were hypomethylation, except for the promoter and 5′UTR, compared to pair-fed mice (Fig. 5B and D). After knowing the global methylation changes, the pattern of HiAlc *Kpn* affecting liver tissue hypomethylation was analyzed. Dnmt3b is a methyltransferase involved in *de novo* synthesis of cytosine methylation. The GO enrichment analysis of downregulated overlap DEGs in liver tissues between W14- and EtOH-fed mice relative to the pair-fed mice showed that *dnmt3b* was decreased in both EtOH- and W14-fed mice, compared to the pair-fed mice (Fig. S1B). The mRNA of *dnmt3b* was verified by RT-qPCR (Fig. 5C), suggesting that HiAlc *Kpn* caused the *de novo* synthesis block of DNA methylation.

The data of DNA methylation and transcriptome in EtOH-fed or W14-fed mice, relative to the pair-fed mice, were shown in visualized upset image (Fig. 5E). Next, the conjoint analysis of the intersecting genes in the GO enrichment pathway and hyper- or hypomethylation gene element regions (promoter or gene-body) in EtOH-fed or W14-fed relative to the pair-fed mice was performed. The data revealed that the GO pathways enrichment of promoter hypomethylation was associated with the significantly upregulated DEGs, while the gene-body hypomethylation was associated with the downregulated DEGs in EtOH-fed mice, compared to the pair-fed mice (Fig. 5F and G). In addition, compared to the pair-fed mice, the GO pathways enrichment of the promoter hypermethylation was associated with the significantly downregulated DEGs, while the gene body, except for 5’UTR hypomethylation, was associated with downregulated DEGs in W14-fed mice (Fig. 5H and I). To verify the correlation between DNA methylation and transcriptome, genes, including *Linp1*, *Rbp4*, *Arntl1*, and *Rora* were selected for methylation-specific PCR (MSP) and RT-qPCR analyses. The results revealed that *Linp1* and *Rbp4* were hypomethylated in the promoter and upregulated in the EtOH-fed mice, compared to the pair-fed mice. Simultaneously, *Arntl1* and *Rora* were hypomethylated in the gene body and downregulated in the W14-fed mice, compared to the pair-fed mice (Fig. 5J and K). Taken together, the data suggest that W14 might induce changes in DNA methylation and regulated gene expression, thereby affecting the progression of NAFLD induced by HiAlc Klebsiella (Fig. 5L).

## DISCUSSION

NAFLD is a chronic liver disease that precedes steatohepatitis, hepatic fibrosis, and cirrhosis. The pathogenic factors of NAFLD include genetics (16, 17), metabolism (18, 19), and microbiome (20), although the existing relationships are not clear. We have previously reported that alcohol-tolerant strains of K. pneumoniae are able to induce NAFLD-like changes in mice (5), in which HiAlc Klebsiella induced liver injury, including fat deposition, ballooning lesions, and changes in the biochemical indicators. The strain of HiAlc *Kpn* W14, isolated from a NASH patient in 2019, could colonize and cause changes in thestructure of bacterial flora in the host intestine (Fig. S2). Additionally, HiAlc *Kpn* might cause changes of immunity and metabolic responses in host. For example, the infiltrating immune cells of T cells, neutrophils, macrophages, and B cells in the livers, and the change of metabolism in the intestine from host induced by HiAlc *Kpn* infection were increased in our previous study (5, 21). However, the mechanism of liver damage in NAFLD mice induced by HiAlc *Kpn* remains unclear. It has been known that aberrant DNA methylation is associated with human cirrhotic liver and hepatocellular carcinoma (10). To explore the potential mechanisms for further understanding the role of DNA methylation in the liver damage of HiAlc *Kpn*-induced NAFLD, we conjointly analyzed methylation and transcriptome levels in the established murine models. Unsurprisingly, our data indicated that the HiAlc *Kpn* induced liver injury, which is consistent with the findings in a previous study (5).

Our data showed that HiAlc *Kpn* induced DNA hypomethylation in liver tissues, which was similar to EtOH-fed mice. Previous studies have shown that, ethanol, as an environmental factor, involved in disease processes by affecting epigenetic changes (22). DNA methylation is a common epigenetic approach, with major concerns reported in several diseases, including cancer. DNA methylation is involved in many cellular processes, including the gene transcription and expression regulation. In addition, DNA methylation is influenced by many known and unknown factors, which was a dynamic schema. Previous studies on whole-genome hypomethylation have been largely overlooked, with most studies focusing on gene-specific hypermethylation events that occur concurrently. However, in recent years, more and more attention has been paid to whole-genome hypomethylation studies. Aberrant epigenetic changes influence the progression of metabolic diseases, including obesity and type-2 diabetes mellitus, by increasing oxidative stress and insulin resistance, while reducing genomic stability (23–25). NAFLD, as a kind of metabolic disease, is induced by HiAlc *Kpn* through the production of endogenous ethanol. Thus, a panorama of DNA methylation and transcriptome analysis in liver tissues of HiAlc *Kpn-* induced NAFLD mice were of great significance in this study.

The transcriptome analysis in our data showed that W14 induced hepatic changes in key gene expression, including *Cyp2e1*, *Acat1*, *Pck1*, *Gata4*, *BMP4*, and *KIF4*, which might enhance liver damage in NAFLD mice model induced by W14. Previous studies have shown that gluconeogenic enzyme PCK1 (26, 27) and Acat1 (28, 29) contributes to lipogenesis in hepatocellular carcinoma. In addition, the expression of monooxygenase CYP2E1 is positively correlated with alcohol-induced liver damage by enhancing the accumulation of toxic intermediate metabolite ROS, which promotes cellular injury and apoptosis (30). On the one hand, the upregulation of *Cyp2e1*, *Acat1*, and *Pck1* in liver tissues of the W14-induced NAFLD mice model suggested that W14 could induce adipogenesis and liver damage. On the other hand, the downregulation of *BMP4* and *KIF4* in liver tissues of the W14-induced NAFLD mice model also induced liver damage. As reported, BMP4 contributed to antisenescent, antisteatotic, antiinflammatory, and antifibrotic responses, while Gremlin 1, an inhibitor of BMP4, is particularly highly expressed in human visceral fat, which regulates hepatic cell senescence during the clinical progression of NAFLD/NASH (31), which is consistent with the findings in this study. The simultaneous depletion of *KIF4* and condensing lead to a complete loss of chromosome morphology (32), inducing DNA injury. In conclusion, the transcriptome analysis proved that W14 induced hepatic changes in key gene expression, which enhanced lipogenesis, or induced the DNA damage in NAFLD mice model induced by W14.

The data of DNA methylation and transcriptome showed that the gene promoter region was hypomethylated, with upregulation of gene expression of *Linp1* and *RBP4* in EtOH-fed mice. Previous studies have shown that *Linp1* is one of the key genes in adipogenesis (33), while *RBP4* is elevated and associated with inflammation in metabolic diseases (34). However, our data showed that these genes were not significantly differentially expressed in the promoter region of W14-fed mice. This may be due to the changes brought about by the bacteria themselves in addition to the alcohol metabolites. In addition, the gene bodies regions were hypomethylated, with downregulation of gene expression of *Arntl1* and *Rorα* in W14-fed mice. As reported, the disruption of the circadian gene *Arntl1* (*Bmal1*) could upregulate the enzymes involved in the *de novo* lipogenesis in epididymal white adipose tissue (35). In addition, RORα plays a significant role in regulating hepatic lipid homeostasis by negatively regulating PPARγ. As a result, the liver-specific *Rorα*-deficient mice develop hepatic steatosis, obesity, and insulin resistance when fed on a high-fat diet (36). Thus, the important regulators of various biological processes, including *Rorα* and *Arntl1*, are disorders regulated by DNA methylation, which plays an important role in regulating the lipogenesis or maintaining the lipid homeostasis in NAFLD induced by W14.

In conclusion, W14 induces DNA hypomethylation in liver tissues and regulates lipid formation and DNA damage associated gene expression. DNA hypomethylation plays an important role in promoting the progression of W14-induced NAFLD. Therefore, a comprehensive understanding of DNA methylation in W14-induced NAFLD is crucial in identifying novel mechanisms between healthy and diseased conditions. The analysis of the DNA methylation landscape contributes to our understanding of the entire disease process, which might be crucial in developing treatment strategies.

## MATERIALS AND METHODS

### Animals and bacterium.

All animal experiments were approved by the Capital Institute of Pediatrics Animal Care and Use Committee on the Ethics of Animal Experiments (permission no. DWLL2021009) and were in accordance with the NIH Guidelines for the Care and Use of Laboratory Animals. Male C57 BL/6N wild-type (WT) mice (6 to 8 weeks old) were purchased from the Beijing Vital River Laboratory Animal Technology Co., Ltd. (Beijing, China). The strain of HiAlc *Kpn* W14 isolated from an ABS/NASH patient in 2019 was used for establishing animal models (5).

### Construction of the murine models of NAFLD.

The animal models were established according to the method previously reported by our group (5). Briefly, WT mice were fed for 1 week to adapt to the environment, then randomly divided into three groups and followed by gavage once every 2 days: HiAlc *Kpn* W14-fed group was gavaged with a single dose of the strain of HiAlc *Kpn* W14 suspended in YPD medium (10^7^ CFU, 200 μL); EtOH-fed mice gavaged with ethanol (40%, 200 μL); and pair-fed mice gavaged with YPD medium (200 μL) were used as positive and negative controls, respectively. In this case, 100% of mice survived after feeding with strains or ethanol. The mice were euthanized after 8 weeks post gavage. The number of animals for each subpanel was ≥ 6, while number of experiments was ≥ 3.

### Histology and physiological assays in the experimental mice.

Mice were euthanized after 8 weeks post gavage. The liver and serum were collected for various analyses. Parts of harvested livers (for hematoxylin and eosin [H&E] staining) were fixed in 10% formalin, embedded, and cut into sections for histological (H&E, Oil Red O) and immunohistochemical staining, while the rest liver tissues and serum were prepared for measuring relevant indices, including ALT, AST, and GSH, to assess the liver injury.

### Genomic DNA extraction.

Genomic DNA (gDNA) extraction of liver tissue was performed using the FastPure cell/tissue DNA isolation minikit (Vazyme, DC102) according to the manufacturer's protocol. Briefly, 20 mg of liver tissue was homogenized in 230 μL lysis buffer (genomic DNA lysis solution A (GA)) and 20 μL proteinase K by a tissue homogenizer. Then the mixture was incubated overnight at 55°C. After centrifuging for 5 min at 13,000 *g*, the supernatant was transferred into a new tube containing 250 μL buffer genomic DNA extraction solution B (GB) and incubated 10 min at 70°C. DNA pellet was precipitated by adding 180 μL of 100% ethanol. gDNA columns were put into collection tubes, while the solution (including the precipitate) was transferred to the adsorption column. The filtrate was discarded after centrifuging for 1 min at 12,000 *g*, while the adsorption column was placed into a collection tube containing 500 μL washing buffer A and 650 μL washing buffer B, respectively. After centrifuging for 1 min at 12,000 *g*, the adsorption column was placed into a new tube containing the preheated 30 μL of elution buffer and incubated for 3 min. After centrifuging for 1 min at 12,000 *g*, eluted gDNA was collected and stored at −80°C until usage.

### Dot blot.

After quantification by Qubit, gDNA (200 ng, 76.5 μL) was denatured in 10 × NaOH (8.5 μL) for 10 min at 95°C, neutralized with 2 M NH_4_OAc (pH 7.0) on ice, and then diluted 2-fold with DNA/RNA enzyme free water. The prepared samples of gDNA (85 μL each) were spotted on an N+ nylon membrane. The blotted membrane was baked at 80°C for 2 h, incubated with 5% milk in PBST for 1 h to block nonspecific antibody binding. After washing with PBST, membrane was incubated with a rabbit anti-5-methylcytosine (5-mC) monoclonal antibody (1:3,000) in PBST at 4°C overnight. After washing for three times with PBST, membrane was then incubated with a secondary antibody (HRP-conjugated sheep antirabbit IgG, 1:4,000) in PBST for 1 h at room temperature. After washing with PBST, membrane was incubated with the enzyme substrate for 1 min. Positive stainings were quantified using a Bio-Rad scanner.

### RNA isolation and construction of RNA-seq libraries.

The total RNA was extracted using TRIzol by FastPure cell/tissue total RNA isolation kit (Vazyme) according to the manufacturer's protocol. And RNA was quantified by Qubit (Invitrogen). Then, the VAHTS Universal V8 RNA-seq library prep kit for Illumina (Vazyme, NR605, China) was used to construct RNA-seq libraries of mRNA enrichment according to the manufacturer’s instructions.

### Real-time qPCR.

Briefly, total RNA was extracted using TRIzol by FastPure cell/tissue total RNA isolation kit (Vazyme). Reverse transcription and cDNA amplification were performed by using a GoScript reverse transcription system (Promega, A5001) and the SYBR green master mix kit (TaKaRa). The primers used are listed in Table 1. GAPDH was used as an internal control for the analysis and normalization of gene expression data sets. The ΔCT values were used for statistical analysis. The data are expressed as the mean values of duplicate real-time qPCR analyses.

**TABLE 1:** Primers of genes used in real-time qPCR

Genes	F-primer sequence (5′–3′)	R-primer sequence (5′–3′)
*cyp2e1*	CGTTGCCTTGCTTGTCTGGA	AAGAAAGGAATTGGGAAAGGTCC
*acat1*	CAGGAAGTAAGATGCCTGGAAC	TTCACCCCCTTGGATGACATT
*pck1*	CTGCATAACGGTCTGGACTTC	CAGCAACTGCCCGTACTCC
*Bmp4*	TTCCTGGTAACCGAATGCTGA	CCTGAATCTCGGCGACTTTTT
*Kif4*	AGGTGAAGGGGATTCCCGTAA	AAACACGCCTTTTATGAGTGGA
*gata4*	CCCTACCCAGCCTACATGG	ACATATCGAGATTGGGGTGTCT
*dnmt3b*	CCTGTGGAGTTTCCGGCTAC	GACGCTCTTAGGTGTCACTTC
*gapdh*	AGGTCGGTGTGAACGGATTTG	TGTAGACCATGTAGTTGAGGTCA

### Whole-genome bisulfite sequencing (WGBS) and DNA methylation data analysis.

Bisulfite conversion was performed using the EpiArt DNA methylation bisulfite kit (Vazyme, EM101-01) according to manufacturer's protocol. DNA library construction was performed after quality testing. The library was sequenced using the NovaSeq platform (PE150) and obtained an average sequencing depth of 30 G reads per sample, then WGBS data were analyzed with a R package. Regions with differences of methylation values >0.2 to the compared group were defined as differential methylation regions (DMRs). This analysis method was used to assessed differentially methylated CpG site in NAFLD induced by HiAlc *Kpn*.

### Bisulifite-specific PCR.

Primers of differentially methylated regions (DMRs) of *Rbp4*, *Linp1*, *Arntl1*, and *Rora* were designed by MethPrimer 1.0 algorithm (http://www.urogene.org/cgi-bin/methprimer/methprimer.cgi) and are shown in Table 2. The PCR system was done using EpiArt HS *Taq* Master Mix (Vazyme, EM202-01, China) according to the manufacturer’s instructions. The PCR product was visualized with agarose gel.

**TABLE 2:** Primers of genes used in methylation-specific PCR

Genes	F-primer sequence (5′–3′)	R-primer sequence (5′–3′)
*Rbp4*	GTTTTTTAGGATAAGTAGAGGACGG	AAACCAACAACTCAAATAAAACGTA
*Linp1*	AGGTGTGTTGGTAAAGTATATACGA	AAAAAAACAAAATTCCACGAC
*Arntl1*	GAATATTTTATTGTTTTTGTAAATCGT	AAATCTAAACTCAAATTCTCTCGCT
*Rora*	TTTGGAGGATAAGGTAGAGAGATTC	CTACCAACCTCCTCTAAAACTACGA

### RNA-seq data analysis.

Total RNA of liver was extracted using FastPure cell/tissue total RNA isolation kit (Vazyme, RC112-01) according to manufacturer's protocol. Sequencing was performed after final library construction. R-package DEseq2 (v.1.36.0) was used to analyze the differentially expressed genes. Significantly differentially expressed genes between any two groups were identified according to the following thresholds: log_2_ (FoldChange)| ≥1 and padj ≤ 0.05.

### Functional enrichment analysis.

ClusterProfiler was used to analyze gene ontology (GO), which visualized functional profiles (GO or KEGG) of genes and gene clusters. For GO functional enrichment analysis, the GO term with *P* < 0.05 was used as the threshold for statistically significant.

### Statistical analysis.

The data were shown as the mean ± the standard deviation (S.D.). All data were compared by analysis of variance (one-way ANOVA) and Student's *t* test using GraphPad Prism 5 (GraphPad Software, USA). *P* < 0.05 was considered statistically significant.

### Limitations of study.

The study has demonstrated that the DNA methylation level can regulate the expression of the key genes, and further to influence the disease processes. However, the current work focuses on detecting DNA methylation in liver tissues of NAFLD mouse induced by W14. For it to be applied in clinical monitoring, more verification work, including data on postintervention reversal, is needed.

### Data availability.

The raw sequence data reported in the present study have been deposited in the Genome Sequence Archive in National Genomics Data Center, Beijing Institute of Genomics (China National Center for Bioinformation), Chinese Academy of Sciences, under accession number CRA009240 and are publicly accessible at https://bigd.big.ac.cn/gsa.
