# Supplementary material for: Combined Methylation and Transcriptome Analysis of Liver Injury of Nonalcoholic Fatty Liver Disease Induced by High Alcohol-Producing Klebsiella pneumoniae
Source: Microbiol Spectr. 2023 Apr 6;11(3):e05323-22. doi: 10.1128/spectrum.05323-22 (PMC10269619; doi:10.1128/spectrum.05323-22)
Supplement: Supplemental file 1 — Fig. S1 and S2. Download spectrum.05323-22-s0001.pdf, PDF file, 0.4 MB [file spectrum.05323-22-s0001.pdf]

Supplemental Figure S1 and Figure S2

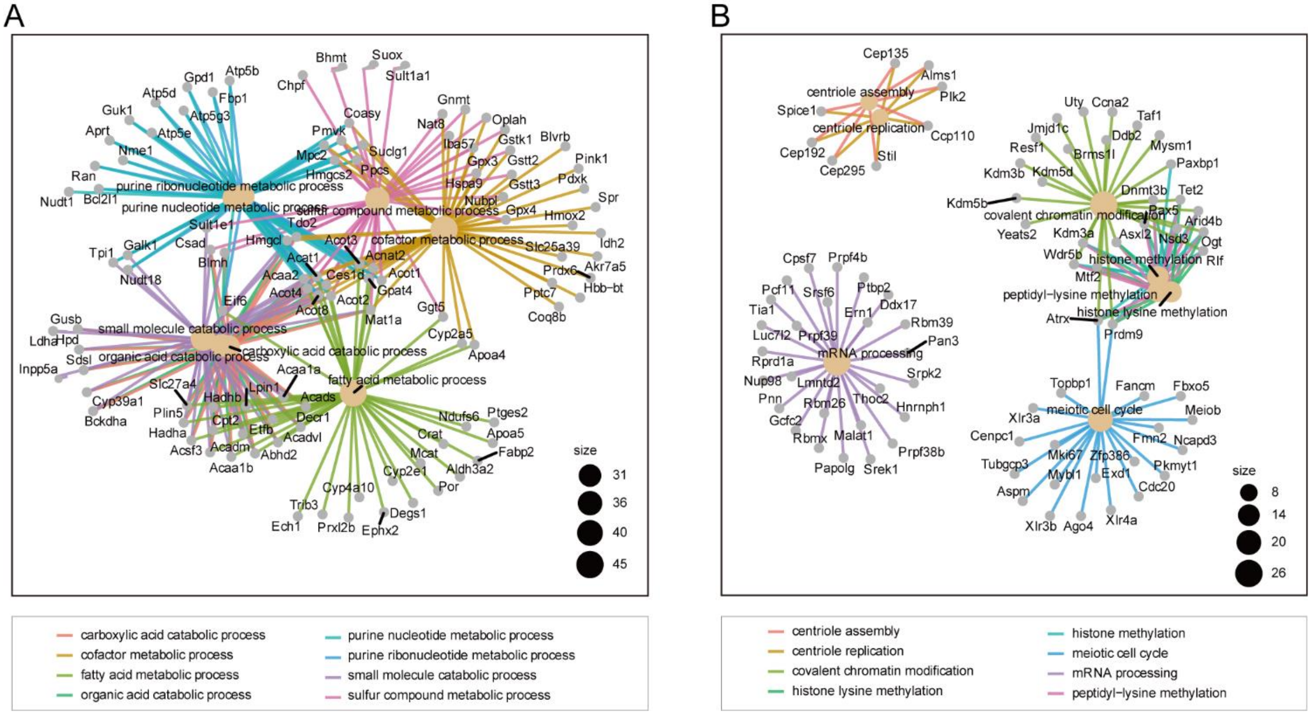

Fig.S1. GO enrichment analysis of up-regulated overlap **(A)** and down-regulated overlap **(B)** DEGs in liver tissues between W14- and EtOH-fed mice relative to the pair-fed mice.

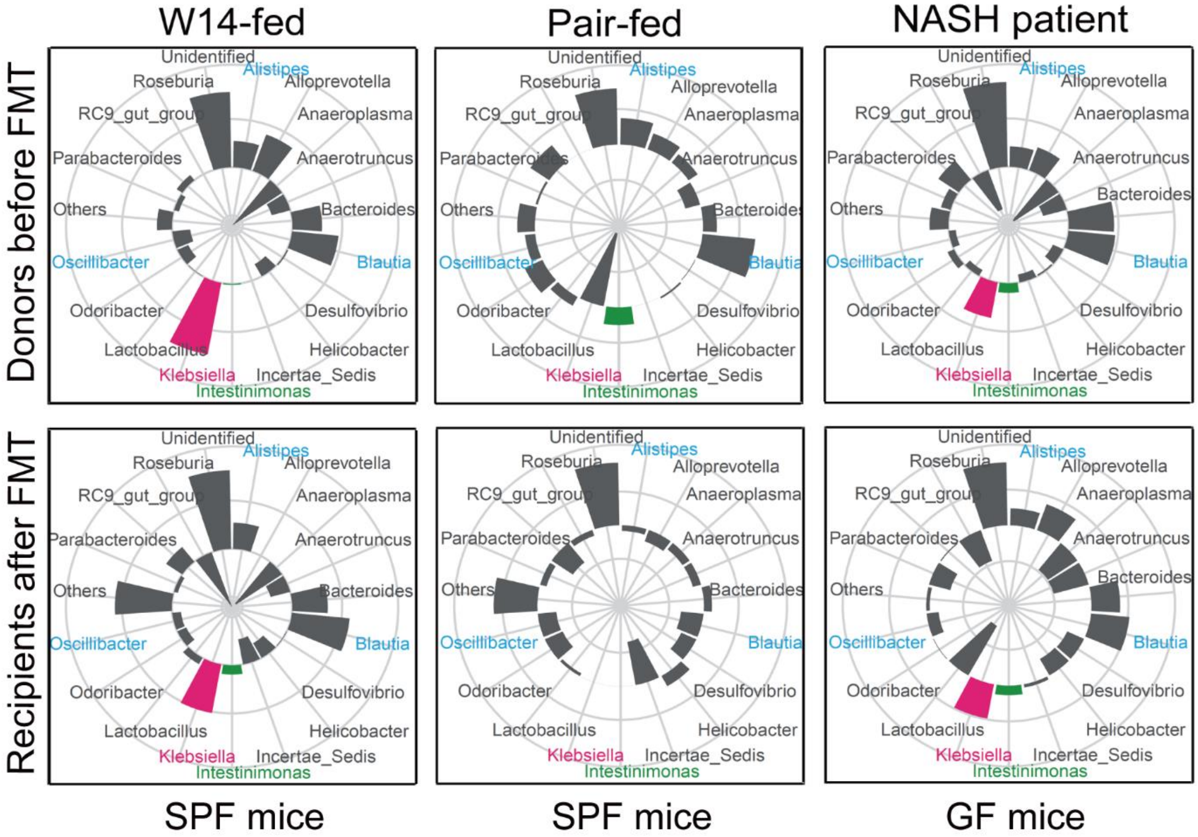

Fig.S2. Analysis of microbiota structure in host intestine before and after fecal microbiota transplantation (FMT). The fecal samples of the patient were transplanted to the germfree mice. HiAlc *Kpn* from the donors to SPF mice.
